# Med-peds rheumatology in practice: assessment after twenty years

**DOI:** 10.1186/1546-0096-10-S1-A17

**Published:** 2012-07-13

**Authors:** Barbara E Ostrov, Lisabeth V Scalzi

**Affiliations:** 1 Pennsylvania State Hershey Medical Center, Hershey, PA, USA

## Purpose

Little is known about the long-term status of pediatric rheumatology patients who transition to adult care. Increasing numbers of Med-Peds graduates have chosen to train in dual subspecialties such as rheumatology. These practitioners are able to provide continuity of care to such patients. A Med-Peds rheumatologist (BEO) has practiced at our medical center providing pediatric rheumatology services for Central Pennsylvania since 1991. Pediatric rheumatology clinics are also performed at outreach sites up to 100 miles away from the home institution, providing care in local communities. Pediatric patients who finish school may see the Med-Peds rheumatologist in the adult practice at the home institution, if they wish. Those who transition and remain with the Med-Peds rheumatologist in the internal medicine-rheumatology practice are the subject of this analysis.

## Methods

The charts of all active patients seen from July 2009 through December 2010 in the adult rheumatology practice followed by the Med-Peds rheumatologist were reviewed. Diagnoses, duration of care, age and gender of patients, and distance traveled to the home institution for appointments was calculated. In a subset of patients with JIA and RA, the number of canceled or no-show appointments was tallied as a surrogate of adherence with care. Chi square and T-tests were used to analyze differences.

## Results

There were 509 individual patients actively followed during the study period. 105 were former pediatric patients (FPP) and 404 patients had always been followed in the adult practice (AAP). The average age of the FPP was 26.7 ± 5.2 and the AAP were 55.7 ±12.4 years. There was no difference in gender with about 15% males in both groups. The duration of follow up was longer for the FPP (12.4 ± 4.4 years versus 11.1 ± 3.3 years; p< .0006). The distance traveled for visits was greater for the FPP (41.2 ± 32.2 versus 26.6 ± 23.9 miles; p < .0001). A greater proportion of patients followed for primary and/or secondary non-inflammatory diagnoses (ex: fibromyalgia, osteoarthritis or osteoporosis) were in the AAP group (p < .00001), although the percentage with primary inflammatory disorders was similar between the groups. In the subset analysis concerning number of office visits missed, there was no difference between the groups.

## Conclusion

About 20% of the active patients in the internal medicine-rheumatology practice of the Med-Peds rheumatologist followed during the 20^th^ year of practice are FPP. These individuals tended to have a lower proportion of non-inflammatory disease, owing to the large number of osteoarthritis patients in the AAP. The FPP traveled further and continued care longer, confirming their preference to maintain continuity with the Med-Peds rheumatologist. The sub-analysis of RA and JIA patients did not reveal differences in visit compliance. Further assessment of adherence and treatment is needed to better understand this population.

## Disclosure

Barbara E. Ostrov: None; Lisabeth V. Scalzi: None.

